# Colorectal Cancer Screening Uptake: Differences Between Rural and Urban Privately-Insured Population

**DOI:** 10.3389/fpubh.2020.532950

**Published:** 2020-11-19

**Authors:** Mesnad Alyabsi, Jane Meza, K. M. Monirul Islam, Amr Soliman, Shinobu Watanabe-Galloway

**Affiliations:** ^1^Population Health Research Section, King Abdullah International Medical Research Center (KAIMRC), Riyadh, Saudi Arabia; ^2^King Saud bin Abdulaziz University for Health Sciences, Riyadh, Saudi Arabia; ^3^Department of Biostatistics, Nebraska Medical Center, College of Public Health, University of Nebraska Medical Center, Omaha, NE, United States; ^4^Department of Epidemiology, Nebraska Medical Center, College of Public Health, University of Nebraska Medical Center, Omaha, NE, United States; ^5^Community Health and Social Medicine, City University of New York School of Medicine, New York, NY, United States

**Keywords:** healthcare disparities, screening, colorectal cancer, geography, private insurance

## Abstract

Earlier studies investigated rural-urban colorectal cancer (CRC) screening disparities among older adults or used surveys. The objective was to compare screening uptake between rural and urban individuals 50–64 years of age using private health insurance. Data were analyzed from 58,774 Blue Cross Blue Shield of Nebraska beneficiaries. Logistic regression was used to assess the association between rural-urban and CRC screening use. Results indicate that rural individuals were 56% more likely to use the Fecal Occult Blood Test (FOBT) compared with urban residents, but rural females were 68% less likely to use FOBT. Individuals with few Primary Care Physician (PCP) visits and rural-women are the least to receive screening. To enhance CRC screening, a policy should be devised for the training and placement of female PCP in rural areas. In particular, multilevel interventions, including education, more resources, and policies to increase uptake of colorectal cancer screening, are needed. Further research is warranted to investigate barriers to CRC screening in rural areas.

## Introduction

Colorectal cancer (CRC) is ranked the third most common cancer in both women and men of the United States (1, 2). The disease develops as a result of polyp development in the colon; though benign initially, the tumor develops into a malignancy within 10 years (3–5). Past CRC research suggests that screening reduces both the incidence and mortality rate by detecting polyps or tumors at a precancerous stage (6, 7). For average-risk individuals who are age-eligible for screening (50–75 years old), the “United States Preventive Service Task Force (USPSTF)” recommends the following screening tests: (1) annual high-sensitivity fecal occult blood test (FOBT); (2) sigmoidoscopy every 5 years with a fecal blood test every 3 years; or (3) colonoscopy every 10 years (8). Despite the demonstrated effectiveness of these screening tests, CRC screening rates remain less than expected. During 2015, only 62.6% of screen-eligible Americans received one of the recommended screening tests, which is lower than the 80% target set by the “Centers for Disease Control and Prevention's” Colorectal Cancer Program (9). Americans 50–64 years old were among the least screened individuals (10).

Several factors predict CRC screening uptake. Factors investigated are age, race, socioeconomic status (SES), availability of insurance, screening cost, accessibility to a usual source of care, communication with provider, level of awareness about CRC screening, perceived colonoscopy pain, rural living, and geographic access to screening facilities (11–16). Despite the modest increase in CRC screening during the past years, individuals who are 50–64 years old, live in rural areas with low income, less education, and lack health insurance were the groups with the lowest increase in CRC screening (9, 17, 18). For example, 71% of individuals aged ≥65 years reported getting screened in 2015, but only 57% in the 50–64 year age group were screened (10, 19). Additionally, analysis of the “Behavioral Risk Factor Surveillance System” (BRFSS) showed that rural populations are unlikely to receive the same level of CRC screening compared to urban populations. Additional studies, including a study from Nebraska, had similar findings (16, 17, 20, 21).

The evidence related to the association between living in rural areas and CRC screening among the 50–64 years old, privately insured population who lives in a rural state is not clear. This unique rural, privately insured population under age 65 is assumed to have “financial access” to screening services. Additionally, the private health insurance population consists mainly of professional individuals who work full time, which is an ideal population. Unlike an older population, busy professionals are more likely to be sensitive to traveling (22, 23).

One-fifth of the U.S. population lives in rural locations, in comparison with 35% rural residents in Nebraska (24, 25). CRC screening in Nebraska has been below the national average. The state ranks 37th nationally in CRC screening, with only 65% of individuals 50–57 years old screened (9). While the screening rate for Nebraskans aged ≥65 years was 72%, the screening rate for individuals between 50–64 years was only 60% (26). In addition to the differences due to age (27, 28), geographic location is a determinant factor for CRC screening. Compared with the urban population, the rural population was 60% less likely to undergo any CRC screening and 57% less likely to undergo colonoscopy screening than their urban counterpart (16). In addition, the use of preventive services and Primary Care Physician (PCP) (PCPs are general practitioners, internists, obstetric gynecologist, and family practitioner) visits are lower in a rural population (27, 29, 30). Combined, individuals who are 50–64 years old and rural residents are the least populations to have CRC screening.

Previous research was based on self-report surveys (e.g., NHIS) or older adults (e.g., Medicare beneficiaries). The current study was designed to evaluate CRC screening uptake among a privately insured population in a rural state. We hypothesize that FOBT use is higher and colonoscopy use lower in rural population compared to the urban population after adjusting for covariates. In addition, we hypothesize that the urban population would have a higher number of PCP visits, and individuals with more PCP visits would be more likely to receive CRC screening. We also investigated the annual use of the FOBT-test by rural-urban status. Elucidating rural-urban disparities would assist policymakers in increasing the rates of colorectal cancer screening among rural residents.

## Materials and Methods

### Data Sources

The data source has been described previously (31). A retrospective cohort study was conducted using data from the Blue Cross Blue Shield of Nebraska (BCBSNE). Blue Cross Blue Shield of Nebraska is a large private health insurer covering more than 700,000 individuals in the state of Nebraska (32). Data consists of claims from inpatient, professional services, and outpatient facilities and comprise codes for disease diagnosis and procedures, of the service date, and the ZIP code of the provider. Members' demographic variables such as age, gender, and ZIP code were all retrieved. The beginning and end dates of coverage were also captured by BCBSNE.

### Study Population

**Figure 1:**
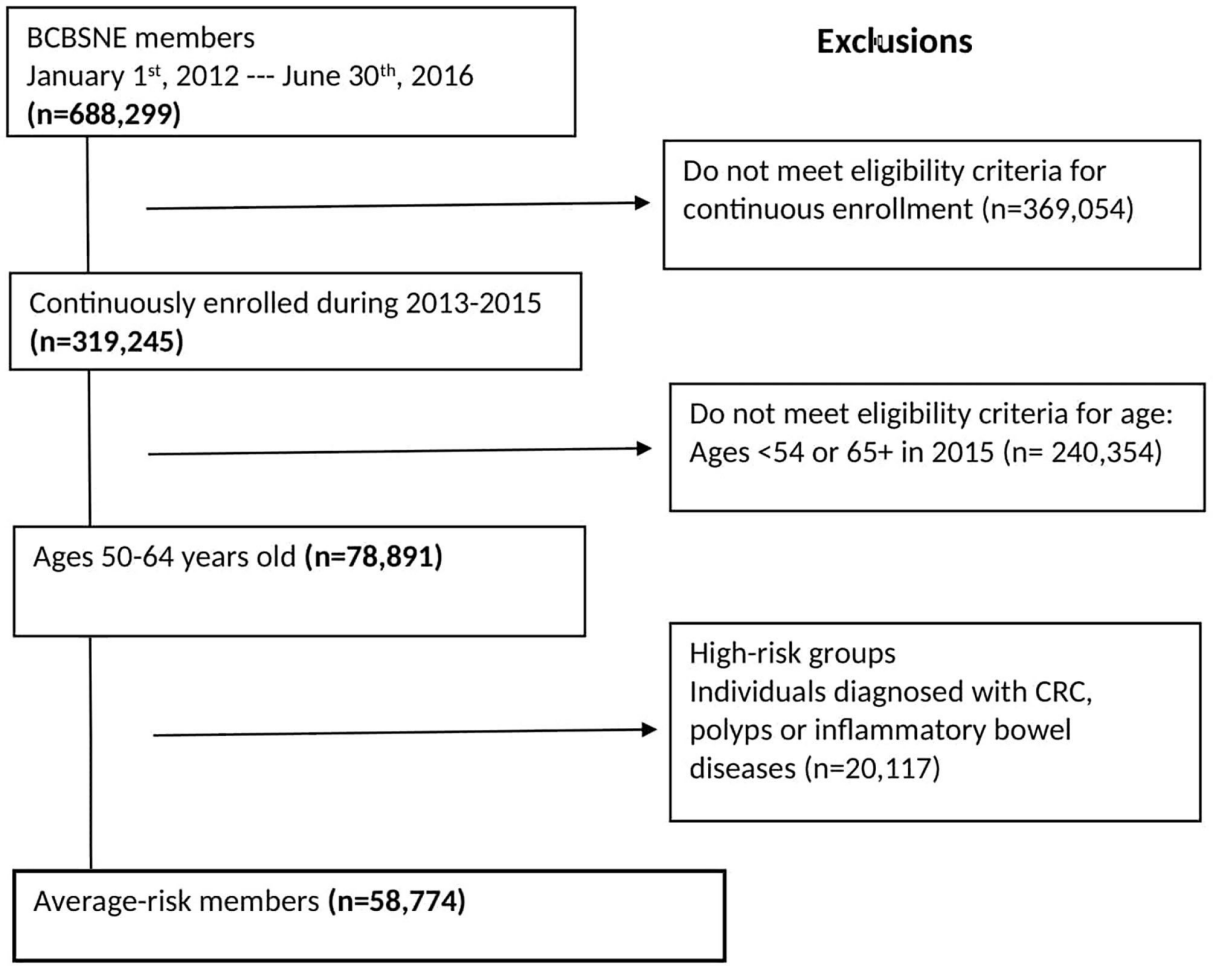
Eligibility criteria for the study population.

Participants were beneficiaries of BCBSNE who were continuously enrolled in the plan from January 1st, 2013 to December 30th, 2015, 50–64 years old, and not diagnosed with CRC, polyps, or inflammatory bowel diseases. We excluded beneficiaries older than 65 because the data possibly may not contain claims for all of their Medicare-covered health services. We also excluded members with CRC, polyps, or inflammatory bowel diseases (i.e., high-risk groups) because we wanted to focus on members who are more likely to receive a CRC screening and not a work-up for an existing disease (i.e., screen-eligible population) (33). Figure 1 shows the eligibility conditions and the number of patients excluded for each condition. The detailed International Classification of Diseases, 9th Revision, Clinical Modification (ICD-9-CM), and 10th Revision (ICD-10-CM) diagnosis codes are shown in Supplementary Figure 1.

### Study Variables

#### Patient Characteristics

The enrollment file was used to extract the beginning and ending dates of coverage and services. The demographics of patients, such as age, gender, and the ZIP code of residence, were derived from the member file. Clinical and test use variables such as FOBT use, colonoscopy use, PCP visits, and the Charlson Comorbidity Index (CCI) were extracted from the claim file using the international classification of disease fields and current procedural terminology (CPT) fields from the claims file.

A PCP visit was defined as an outpatient visit with any of the following types of providers: general practitioner, internist, obstetric gynecologist, and family practitioner. Patients who had at least one outpatient claim associated with a PCP in the calendar years of 2013 or 2014 was considered to have had a PCP visit. The PCP visit was categorized into no visits vs. any visits and 0, 1–2, 3–5, and ≥6 visits.

The CCI was calculated to determine the burden of disease among the study population. To apply the index to administrative data, many authors modified the comorbidity index and validated its use with administrative data. For instance, Quan modifications were used in this study by adjusting the weights of comorbidities (34). Charlson Comorbidity Index was computed during the years 2013 and 2014 and was categorized into 0, 1, and ≥2.

#### Rural-Urban Measurement

“Rural-Urban Community Area Codes” (RUCA) was used to determine members' rural-urban status (35). Rural-Urban Community Area Codes is constructed from census tract and uses the standard “Bureau of Census Urbanized Area” (U.A.) and “Urban Cluster” (U.C.) definition in combination with work commuting information to characterize all of the Nation's census tracts regarding their rural and urban status and their relationship. The classification designates “metropolitan” (i.e., primary commuting flow within a U.A.), “micropolitan” (i.e., large rural or primary flow within an U.C. of 10,000–49,999 individuals), “small town” (i.e., primary flow within an U.C. of 2,500–9,999 individuals) and “rural commuting areas” (i.e., primary flow to a tract outside a U.A. or U.C.) with numbers between 1 and 10. The numbers are segmented into 21 secondary codes depending on commuting flows.

Though the original RUCA classification was based on the census tract, it uses the ZIP code as its geographic unit. The updated RUCA codes are dependent on the 2010 decennial census and the 2006–2010 “American Community Survey.” In the current study, the 33 codes were combined into urban and rural as recommended by the “Washington, Montana, Wyoming, Idaho, and Alaska Rural Health Research Center” (35, 36).

## Outcome Variables

The outcome used in this study was the use of CRC screening during the year 2015. Fecal occult blood test use was defined as the proportion of the eligible population who have ≥1 claim for FOBT during the year. The CPT codes used to identify FOBT uptake were: 82,270, 82,272, and 82,274 (37).

Colonoscopy was defined as the proportion of the study population who have ≥1 claim for a colonoscopy during the year. The following CPT and ICD codes were used to identify colonoscopy uptake: 44,388–44,394, 44,397, 45,355, 45,379, 45,381, 45,386, 45,387, 45,378, 45,380, 45,382–45,385, 45.21–45.23, and 45.25 (37). Because administrative data do not provide information about the indication of colonoscopy (i.e., screening, diagnostic, or surveillance), we were unable to identify the purpose of the test (33, 38, 39). However, to limit the population to members who potentially used screening-colonoscopy, we restricted the sample to those with the following procedural codes that indicate screening: V7651, Z1211, V7641, and Z1212 (40).

### Data Analysis

Age, gender, rural-urban status, number of PCP visits during 2013 and 2014, and the CCI level during 2013 and 2014 were compared between test users (FOBT or colonoscopy) vs. non-users using the Chi-square-test. In the univariate analysis, we compared the FOBT use in 2015 vs. no FOBT use and the colonoscopy use in 2015 vs. no colonoscopy use using logistic regression. To assess the predictor's significance, Wald-tests were used. We reported OR of FOBT screening and the 95% confidence intervals (CI) and OR of colonoscopy screening and the 95% CI. We also fitted two multivariate logistic regression models for FOBT and colonoscopy to evaluate the relationship between rural-urban status and screening use adjusted for age, gender, PCP visits, and CCI.

To estimate the annual prevalence rate for FOBT (years 2012–2016), the numerator was computed as the number of members with at least one paid claim for the specified screening test during the specific year. The denominator was the frequency of members eligible for screening during the specific year. To compare the rates between urban and rural members, we used Chi-square-tests. The same test was used to assess the CRC screening use across years. All tests were 2-tailed, and the chosen α level was 0.05. SAS statistical software version 9.4 (SAS Institute Inc. Cary, NC) was used to perform all analyses. This study was approved by the University of Nebraska Medical Center Institutional Review Board (IRB# 366-1).

## Results

Applying the eligibility condition resulted in a total cohort of 58,774 (Figure 1). Table 1 shows the characteristics of individuals who are eligible for CRC screening by rural and urban status. It also presents the eligible population by age, gender, PCP visits, and CCI in 2013 and 2014. Most of the urban population is female (55%), and the majority of the urban population has visited the PCP during 2013–2014 (80%).

**Table 1:** Characteristics of BCBSNE members eligible for colorectal cancer screening by rural/urban residence (*N* = 58,774).

**Characteristics**	**Rural (*n* = 30,460)**	**Urban (*n* = 28,312)**	***P-*value**
**Age**			
50–54	9,132 (30.0)	9,381 (33.0)	<0.001
55–59	10,959 (36.0)	9,988 (35.0)	
60–64	10,369 (34.0)	8,943 (32.0)	
**Gender**			
Female	16,052 (53.0)	15,477 (55.0)	<0.001
Male	14,408 (47.0)	12,835 (45.0)	
**PCPs visits (2013–2014)**			
Yes	23,122 (76.0)	22,551 (80.0)	<0.001
No	7,338 (24.0)	5,761 (20.0)	
**CCI (2013–2014)**			
0	20,948 (69.0)	18,646 (66.0)	<0.001
1	5,657 (18.0)	5,577 (20.0)	
≥2	3,855 (13.0)	4,089 (14.0)	

**Figure 2:**
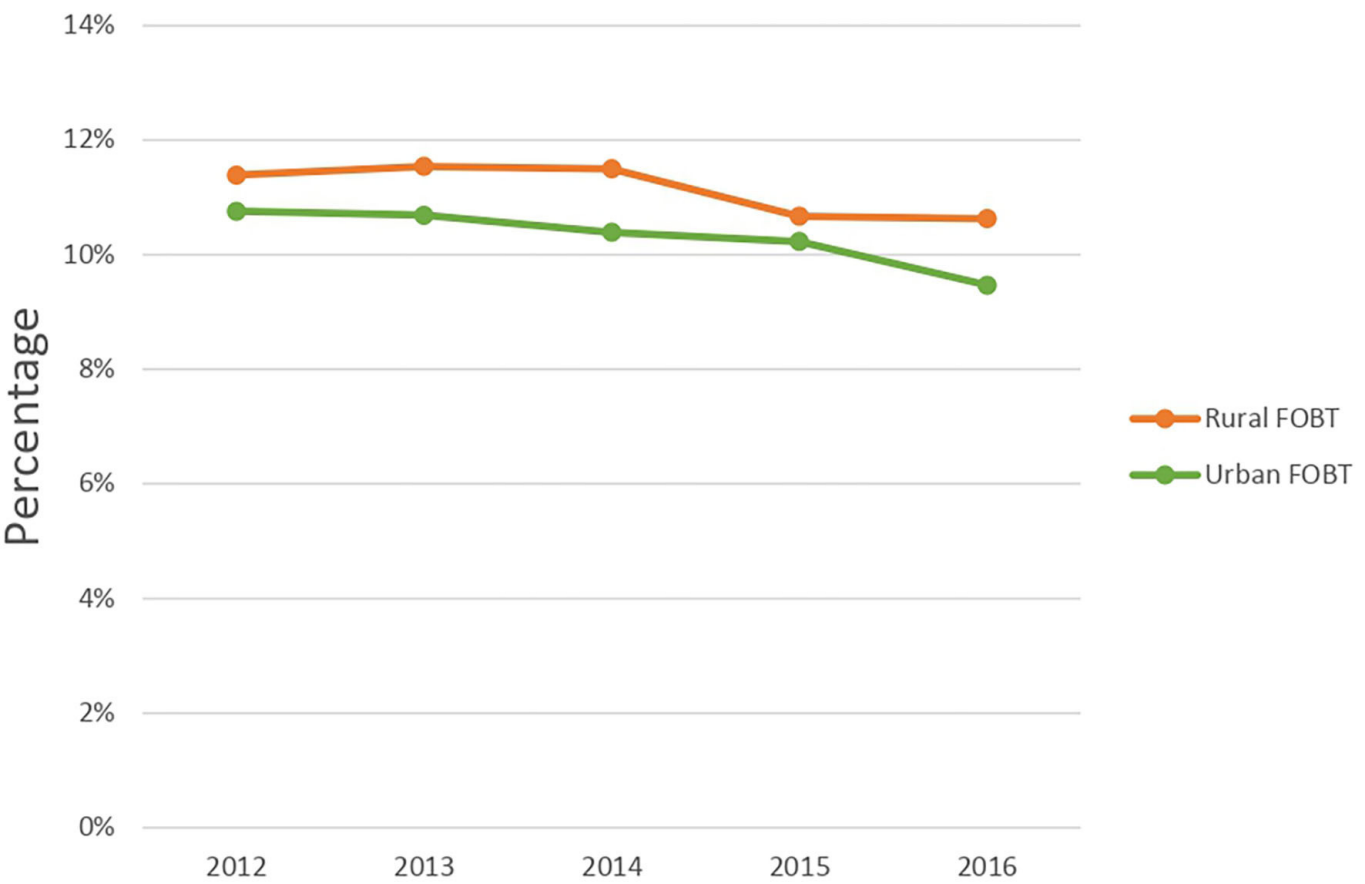
Annual fecal occult blood test in Blue Cross Blue Shield Nebraska population, 2012–2016.

Figure 2 and Supplementary Figure 2 shows the overall annual FOBT use among the rural and urban populations. Between 2012 and 2016, FOBT use was significantly higher in rural residents compared to urban populations. The findings suggest that rural members had a higher proportion of FOBT use compared to urban members (*P* < 0.05). Rural members had a consistently higher FOBT use for age groups 50–54 and 55–59, but this pattern was reversed for the oldest age group of 60–64 years during the year 2016. In rural areas, females had consistently higher use of FOBT than males (e.g., 15% of females used FOBT, while only 7% of males used FOBT during 2012). For both areas, the frequency of PCP visits was proportionally associated with FOBT use.

Table 2 displays predictors of FOBT use in 2015. After controlling for age, gender, CCI, and PCP visits, rural residents had 56% increased odds of receiving FOBT compared to urban populations (OR = 1.56; 95% CI: 1.45, 1.69). Females had 85% increased odds of FOBT use compared to males (OR = 1.85; 95% CI: 1.69, 2.0). For PCP visits, the higher the number of visits, the higher the odds of FOBT use. Overall, the use of FOBT in the past year was 4.2%. We also found an interaction between rural-urban status and gender (Table 3). While rural females had 68% decreased odds of using FOBT during 2015 (OR = 0.32; 95% CI: 0.28, 0.36), urban females had 15% larger odds of using FOBT (OR = 1.15; 95% CI: 1.01, 1.31).

**Table 2:** Univariate and multivariate analyses of variables associated with FOBT screening using logistic regression models, BCBSNE 2013–2015.

	**FOBT use in 2015**				
	**FOBT use**	**No FOBT use**	***P-*value**	**Univariate OR (95% CI)**	**Multivariate OR (95% CI)**
**Age**					
50–54	729 (29.0)	17,785 (32.0)	0.03	1.0	1.0
55–59	891 (36.0)	20,056 (36.0)		1.08 (0.98, 1.20)	1.05 (0.95, 1.17)
60–64	863 (35.0)	18,450 (33.0)		1.14 (1.03, 1.26)	1.08 (0.97, 1.19)
**Gender**					
Male	803 (32)	26,441 (47.0)	<0.0001	1.0	1.0
Female	1,680 (68)	29,850 (53.0)		1.85 (1.69, 2.04)	1.85 (1.69, 2.0)
**Member status**					
Urban	952 (38.0)	27,334 (49.0)	<0.0001	1.0	1.0
Rural	1,531 (62.0)	28,955 (51.0)		1.51 (1.41, 1.67)	1.56 (1.45, 1.69)
**PCP visits in 2013–2014**					
0	396 (16.0)	12,703 (23.0)	<0.0001	1.0	1.0
1–2	646 (26.0)	15,062 (27.0)		1.38 (1.21, 1.56)	1.37 (1.21, 1.56)
3–5	733 (30.0)	14,796 (26.0)		1.59 (1.40, 1.80)	1.59 (1.40, 1.80)
6–9	457 (18.0)	8,490 (15.0)		1.73 (1.50, 1.98)	1.71 (1.49, 1.96)
≥10	251 (10.0)	5,240 (9.0)		1.54 (1.31, 1.81)	1.49 (1.26, 1.75)
**CCI in 2013–2014**					
0	1,929 (78.0)	43,847 (78.0)	0.92	1.0	1.0
1	373 (15.0)	7,299 (15.0)		1.02 (0.91, 1.14)	0.99 (0.87, 1.11)
≥2	181 (7.0)	4,145 (7.0)		0.99 (0.85, 1.16)	0.95 (0.81, 1.11)

**Table 3:** Adjusted association between rural-urban status and FOBT use.

**Rural-urban status**	**Gender**	
	**Male**	**Female**
Rural	1.0	0.32 (0.28, 0.36)
Urban	1.0	1.15 (1.01, 1.31)

*Adjusted for age, gender, Primary Care Physician visits (PCP), and Charlson Comorbidity Index (CCI)*.

The predictors for colonoscopy use in 2015 are shown in Table 4. Although the univariate analysis showed higher colonoscopy use among urban residents (OR = 1.09; 95% CI: 1.01, 1.17), the multivariate analysis showed no significant difference (OR = 1.06; 95% CI: 0.98, 1.14). There is an inverse association between age and the colonoscopy use during 2015. While individuals older than 60 years old had a 14% decreased in odds of colonoscopy use (OR = 0.86; 95% CI: 0.80, 0.93) compared with those <55 years old, individuals between 55–59 years had 44% decreased odds (OR = 56; 95% CI: 0.60, 0.71). Compared with males, females had 16% increased odds in FOBT use (OR = 1.16; 95% CI: 1.09, 1.25). In addition, the higher the frequency of PCP visits, the higher the odds of colonoscopy use.

**Table 4:** Univariate and multivariate analyses of variables associated with the use of colonoscopy using logistic regression models, BCBSNE 2013–2015.

	**Colonoscopy use in 2015**				
	**Colonoscopy use**	**No colonoscopy use**	***P***	**Univariate OR (95% CI)**	**Multivariate OR (95% CI)**
**Age**					
50–54	1,308 (43.0)	17,206 (31.0)	<0.0001	1.0	1.0
55–59	809 (27.0)	20,138 (36.0)		0.53 (0.48, 0.58)	0.56 (0.60, 0.71)
60–64	926 (30.0)	18,387 (33.0)		0.66 (0.61, 0.72)	0.86 (0.80, 0.93)
**Gender**					
Male	1,297 (43.0)	25,947 (47.0)		1.0	1.0
Female	1,746 (57.0)	29,784 (53.0)	<0.0001	1.18 (1.09, 1.26)	1.16 (1.09, 1.25)
**Member status**					
Rural	1,515 (50.0)	28,971 (52.0)	0.02	1.0	1.0
Urban	1,528 (50.2)	26,758 (48.0)		1.09 (1.01, 1.17)	1.06 (0.98, 1.14)
**PCP visits in 2013–2014**					
0	521 (17.0)	12,578 (23.0)	<0.0001	1.0	1.0
1–2	834 (27.0)	14,874 (27.0)		1.35 (1.21, 1.51)	1.36 (1.21, 1.52)
3–5	895 (29.0)	14,634 (26.0)		1.48 (1.32, 1.65)	1.51 (1.35, 1.68)
≥6	793 (26.0)	13,645 (24.0)		1.40 (1.25, 1.57)	1.47 (1.31, 1.65)
**CCI in 2013–2014**					
0	2,415 (79.0)	43,361 (78.0)	0.03	1.0	1.0
1	439 (14.0)	8,233 (15.0)		0.95 (0.86, 1.06)	0.95 (0.85, 1.05)
≥2	189 (6.0)	4,137 (7.0)		0.82 (0.70, 0.95)	0.84 (0.71, 0.97)

*PCP visits, Primary Care Physician visits; CCI, Charlson Comorbidity Index*.

## Discussion

The current study hypothesized that FOBT use is higher, and colonoscopy use is lower in the rural population and that the urban population would have a higher frequency of PCP visits, and individuals with higher PCP visits will be more likely to receive CRC screening. The functions of PCP consist of discussion and suggestions about screening, performing non-invasive screening tests (e.g., FOBT), and referring patients to specialists (e.g., gastroenterologists) who can perform an endoscopic screening test (41). Our study uncovered several significant findings of the differences in the use of CRC screening between the rural and the urban privately insured populations. Firstly, FOBT utilization was significantly decreasing since 2013, among both rural and urban populations (*P* < 0.05). Secondly, multivariate regression analysis indicated that though FOBT use was significantly higher in the rural population, there was no significant difference in colonoscopy use between rural and urban residents. It also showed an interaction between rural-urban status and gender and the use of FOBT. Thirdly, multivariate regression analysis also indicated that the odds of FOBT and colonoscopy uses were higher among females and were positively associated with PCP visits, but colonoscopy use was lower among individuals older than 55 and those with ≥2 CCI.

Overall, we found a decline in FOBT use since 2013; similar findings were reported previously (42–44). While the annual FOBT use was 10–11% is slightly higher than recent rates from national surveys, which was between 5–8% (17, 37, 45), it is comparable to claims-based studies (46). For instance, Ladabaum and colleagues used a sample of 21 million privately insured population and found that ~10% used the annual FOBT-test during 2009 (46). The discrepancy between our findings and results from surveys is possible because their results are based on self-reported data, prone to recall bias, and some of the population surveyed is uninsured or underinsured, which might result in lower screening rates (47, 48). Among the continuously enrolled individuals from 2013 to 2015, we found that 5% of the rural population used FOBT during 2015, similar to findings from a Nebraskan rural study (18).

Furthermore, we found that the rural population is 56% more likely to use FOBT compared with the urban population, similar to a previous study using national data (17), but not to a study using data from Texas (20). The finding is encouraging since it suggests that this privately insured rural population has access to non-invasive screening tests through their PCPs, a test that is linked with a reduction in both the incidence and mortality due to CRC (49). Conversely, it might reflect a shortage of specialists to perform a colonoscopy in rural areas, prompting local PCPs to recommend non-invasive tests (50). If that is the case, training non-specialists to perform colonoscopy is necessary for both screening and follow-up of FOBT-positive individuals. Surprisingly, while the urban population in the current study was significantly more likely to visit PCPs during 2013–2014, rural residents were more likely to use FOBT.

We observed that the rural population was 56% more likely than the urban population to use FOBT, but rural women were 68% less likely than rural men to use FOBT. The result suggests gender disparity in FOBT use. A potential contribution to the disparity in FOBT use is the current characteristic of practicing PCPs in rural Nebraska, pre-dominantly males (85%). Prior literature shows that male PCPs are less likely than female PCPs to practice patient-centered communication or spend more time with the patient during clinic visits to discuss preventive services such as screening (51, 52). Possible targeted interventions that could ameliorate gender disparity in FOBT use among rural women in Nebraska and other similar rural populations would include increasing the supply of female PCPs in rural areas or enhancing male PCPs' awareness about CRC screening that could consist of the implementation of reminder systems.

The univariate analysis shows that urban residents were more likely to use colonoscopy, though the association disappeared in the multivariate model. The non-significant finding has been reported previously using a national sample (20) and confirmed in this privately insured population. This finding is surprising given that all individuals in this study are privately insured, and that the higher rates of uninsured or underinsured in rural areas reported previously in the national survey have no impact on the association between rural-urban status and CRC-test use among our population. In particular, the comprehensive health care reform law that was enacted in March 2010 makes compulsory that private health insurance plans to cover all preventive services suggested by the “United States Preventive Services Task Force” and graded “A” or “B,” which cover CRC screening, with no out-of-pocket costs for members (53). Accordingly, barriers other than health access, which was equal between the rural and urban residents, should be illuminated in future studies, especially factors related to the increased distance to providers or decreased number of specialists in rural areas (54).

Congruent with prior research (33, 55–59), we found that the more frequent the PCP visits, the more likely screening tests will be used. The associations between PCP visits and test receipts remained significant after adjustment for CCI. The result indicates that the discussion between PCP and patients about preventive services such as screening occurs during annual checkups or routine care visits vs. acute visits (60–62). Therefore, not only access to care has an important role in determining whether individuals receive CRC screening test but also the frequency of PCP visits. Lastly, there was an interaction between gender and rural-urban status and the use of FOBT. Urban females were significantly more likely to receive FOBT compared to urban males (50, 63). However, it is unclear why rural females are less likely to use FOBT compared to rural males. It is possible that PCPs in rural areas are less likely to offer females alternative testing when colonoscopy is not possible (50). Alternatively, rural females are at a disadvantage for FOBT uptakes since 85.8% of rural Nebraskans' PCPs are male, and that patients who live in rural Nebraska and saw a male PCPs are 44% less likely to be up to date in CRC screening compared to those who saw a female PCPs (18). Consequently, policies advocating for an increased number of female PCPs in rural areas should be prioritized.

The current study has many strengths. Our cohort consisted of a large sample derived from the largest private health insurer in Nebraska, which has never been studied. Additionally, the private health insurance population consists mainly of professional individuals who work full time, an ideal population because, unlike an older population, busy professionals are more likely to be sensitive to traveling or taking off time to undergo screening. Also, the current result reflects a CRC screening test utilization in the community setting.

The findings should be interpreted with caution. First, about 2% of BCBSNE members ended their membership during December 2014, and this may have slightly impacted the total number of members eligible for CRC screening during the year 2015. Second, we were uncertain about the intent of the colonoscopy-test because it was derived from claim data; nonetheless, we restricted colonoscopy users to individuals with claims indicating screening as previously reported (37, 40). Third, we were limited to 4 years of data, and therefore we were unable to assess the colonoscopy use during the 10-year recommended period for screening colonoscopy suggested by the USPSTF. Extrapolating our colonoscopy use during 2015 to 10-year utilization would result in colonoscopy utilization of 51.8%, an estimate similar to what has been reported during 2015 among 50–64 years old privately insured using national data; 50–62% (45) and to a study from Nebraska, 53% (18). It also resembles findings from national surveys such as NHIS (53–56%) and BRFSS (53.8%) (Table 5). Fourth, the current study is constrained to people who are privately insured and live in Nebraska, and our results are therefore not generalizable to other states, with different types of insurance, the underinsured or the uninsured. Nevertheless, the population of Nebraska represents the population of the U.S. well in their sociodemographic characteristics, with the only difference that Nebraska has a higher percentage of whites than the U.S. average (79.0 vs. 60.7%) (68). Fifth, prior studies show that SES is positively associated with access to health services use (69). In our study, we were unable to adjust for the effect of SES. However, our sample consists of a privately-insured population who we believe is homogenous. We also believe that insurance status as a proxy to SES since it has been found important in predicting health outcomes in terms of access to health care (69).

**Table 5:** Comparison of CRC screening with national and state data (%).

	**Annual FOBT**	**Annual colonoscopy**	**FOBT during past year**	**Colonoscopy during past 10 years**	**Up to date**
Current study	10	14	5	51.8	-
NHIS (6, 19, 45, 64)	10	-	5.9	53–56	56–61
BRFSS (17, 20, 36, 65)	9	26–32	-	53.8	54–65
Other claims data (37, 43, 45, 46, 66, 67)	7.9–10.4	10–20.9	-	53	47.4–63.4
EMRs in Nebraska (18)	-	-	5	53	55

*EMRs, Electronic medical records; NHIS, National Health Interview Survey; BRFSS, Behavior Risk Factor Surveillance System*.

We found that the overall rural population was significantly more likely to use FOBT, but females who live in rural areas are less likely to receive FOBT compared to rural males. This disparity might reflect the characteristics of PCPs practicing in rural areas who are pre-dominantly male and are less likely to recommend screening (18). The value of preparing and placement of female PCPs in rural areas must be a priority since it could contribute to an increase in CRC screening. In particular, providers should be encouraged to discuss CRC screening during patients' regular visits when the use of preventive services in general, and CRC screening, especially, are detailed.

## Data Availability Statement

Data cannot be shared publicly because of sensitive identifier that have been used in this study, which were used under license for the current study. Moreover, we have signed a contract with the BCBSNE in which we have stated that all data are reserved. Data are available for any interested researcher who meets the criteria for access to confidential data from: www.nebraskablue.com.

## Ethics Statement

The University of Nebraska Medical Center Institutional Review Board (IRB# 366-1) approved this study. Written informed consent for participation was not required for this study in accordance with the national legislation and the institutional requirements.

## Author Contributions

MA planned the study, conducted the analyses, and drafted the manuscript. MA, AS, KI, and SW-G helped with the study planning and aided with the manuscript preparation. JM assisted with revising data analysis. SW-G and AS helped with data acquisition. All authors helped with manuscript revisions, read, and approved the submitted manuscript.

## Conflict of Interest

The authors declare that the research was conducted in the absence of any commercial or financial relationships that could be construed as a potential conflict of interest.

## Abbreviations

CRC: Colorectal Cancer
BCBSNE: Blue Cross Blue Shield of Nebraska
RUCA: Rural Urban Commuting Area
ICD: International Classification of Diseases
FOBT: Fecal Occult Blood Test.
