# Supplementary material for: Colorectal Cancer Screening Uptake: Differences Between Rural and Urban Privately-Insured Population
Source: Front Public Health. 2020 Nov 19;8:532950. doi: 10.3389/fpubh.2020.532950 (PMC7710856; doi:10.3389/fpubh.2020.532950)
Supplement: Supplementary file 1 [file Data_Sheet_1.docx]

| **Supplementary Figure 1. High-risk groups excluded from the study samples** | |
| --- | --- |
| **Condition** | **ICD Diagnosis** |
|  |  |
| CRC diagnosis | 153.0,C183,153.1,C184,153.2,C186,153.3,C187,153.4,C180,153.6,C182,  153.7,C185,153.8,C188,153.9,C189,154.0,C19,154.1,C20,154.8,C218. |
|  |  |
| Polyps diagnosis | V1272,Z86010 |
|  |  |
| Inflammatory Bowel Disease | 5551,K5010,5552,K5080,5559,K5090, 5561,K5180,  5562,K5120,5563,K5130, 5565,K5150,5566,K5100,  5568,K5180,5569,K5190,5581,K520,5582,K521, 5589,K5289,K529 |

**Supplementary Figure 2. Annual percentage of FOBT use by rural-urban status, BCBSNE 2012–2016**

|  | 2012  (n=8,527) | | 2013  (n=8,827) | | 2014  (n=8,716) | | 2015  (n=7,870) | | June, 2016  (n=7,229) | |
| --- | --- | --- | --- | --- | --- | --- | --- | --- | --- | --- |
|  |  | |  | |  | |  | |  | |
| Overall by location | Rural  (n=4,706) | Urban  (n=3,821) | Rural  (n=4,827) | Urban  (n=4,000) | Rural  (n=4661) | Urban  (n=4,055) | Rural  (n=4,066) | Urban  (n=3,804) | Rural  (n=3,750) | Urban  (n=3,479) |
|  |  |  |  |  |  |  |  |  |  |  |
| Characteristics | 11.39  (11.09,11.7) | 10.76  (10.44,11.0) | 11.54  (11.24,11.8) | 10.69  (10.37,11.0) | 11.50  (11.19,11.8) | 10.39  (10.09,10.6) | 10.67  (10.36,10.98) | 10.23  (9.92,10.53) | 10.63  (10.31,10.95) | 9.47  (9.17,9.77) |
|  |  |  |  |  |  |  |  |  |  |  |
| Age |  |  |  |  |  |  |  |  |  |  |
| 50-54 | 11.69  (11.16,12.2) | 9.82  (9.31,10.35) | 11.80  (11.29,12.3) | 9.70  (9.21,10.20) | 11.73  (11.21,12.2) | 9.40  (8.92,9.88) | 10.86  (10.34,11.38) | 9.30  (8.82,9.79) | 10.92  (10.39,11.46) | 8.77  (8.30,9.25) |
|  |  |  |  |  |  |  |  |  |  |  |
| 55-59 | 11.95  (11.42,12.4) | 11.47  (10.90,12.0) | 11.94  (11.41,12.4) | 11.35  (10.79,11.9) | 11.82  (11.29,12.3) | 11.01  (10.48,11.5) | 10.98  (10.45,11.51) | 10.84  (10.30,11.38) | 11.01  (10.47,11.56) | 10.13  (9.61,10.67) |
|  |  |  |  |  |  |  |  |  |  |  |
| 60-64 | 10.44  (9.91,10.97) | 11.09  (10.50,11.6) | 10.77  (10.22,11.3) | 11.16  (10.58,11.7) | 10.83  (10.27,11.4) | 10.93  (10.36,11.5) | 10.05  (9.49,10.62) | 10.69  (10.10,11.27) | 9.74  (9.16,10.33) | 9.58  (9.02,10.14) |
|  |  |  |  |  |  |  |  |  |  |  |
| Gender |  |  |  |  |  |  |  |  |  |  |
| Female | 15.0  (14.53,15.4) | 10.99  (10.55,11.4) | 15.18  (14.71,15.6) | 10.91  (10.48,11.3) | 15.18  (14.70,15.6) | 10.61  (10.20,11.0) | 14.34  (13.86, 14.82) | 10.53  (10.11,10.95) | 14.27  (13.76,14.77) | 9.56  (9.16,9.97) |
|  |  |  |  |  |  |  |  |  |  |  |
| Male | 7.34  (6.97,7.70) | 10.47  (10.0,10.95) | 7.47  (7.11,7.85) | 10.42  (9.95,10.88) | 7.40  (7.03,7.78) | 10.12  (9.67,10.57) | 6.52  (6.16,6.88) | 9.85  (9.40,10.30) | 6.53  (6.15,6.90) | 9.36  (8.91,9.81) |
|  |  |  |  |  |  |  |  |  |  |  |
| PCP visit |  |  |  |  |  |  |  |  |  |  |
| Yes | 12.27  (11.92,12.6) | 11.90  (11.54,12.2) | 12.32  (11.98,12.6) | 11.69  (11.34,12.0) | 12.29  (11.94,12.6) | 11.47  (11.14,11.8) | 11.45  (11.10,11.80) | 11.35  (11.01,11.70) | 11.52  (11.16,11.88) | 10.90  (10.55,11.24) |
|  |  |  |  |  |  |  |  |  |  |  |
| No | 6.40  (5.79,7.02) | 2.58  (2.11,3.05) | 6.75  (6.10,7.39) | 2.62  (2.13,3.11) | 6.49  (5.84,7.15) | 2.18 (1.76,2.61) | 6.02  (5.39,6.67) | 1.97  (1.56,2.38) | 5.63  (5.01,6.25) | 1.40  (1.09,1.71) |
