# Supplementary material for: Colorectal Cancer Screening Uptake: Differences Between Rural and Urban Privately-Insured Population
Source: Front Public Health. 2020 Nov 19;8:532950. doi: 10.3389/fpubh.2020.532950 (PMC7710856; doi:10.3389/fpubh.2020.532950)
Supplement: Supplementary file 2 [file Table_1.docx]

| Supplementary Table 1. Frequencies and Percentages of BCBSNE and The State of Nebraska Population by Regional Health Department | | | | | |
| --- | --- | --- | --- | --- | --- |
| Region | BCBSNE members | | The State of Nebraska | | |
|  | N | % | N | | % |
| Panhandle Public Health District | 25731 | 3.74 | 88403 | 4.85 | |
| North Central District Health Department | 19938 | 2.90 | 46394 | 2.54 | |
| Northeast Nebraska Public Health Department | 10331 | 1.50 | 31387 | 1.72 | |
| Dakota County Health Department | 2656 | 0.39 | 21006 | 1.15 | |
| West Central District Health Department | 1584 | 0.23 | 39433 | 2.16 | |
| Loup Basin Public Health Department | 14229 | 2.07 | 31140 | 1.71 | |
| East Central District Health Department | 21093 | 3.06 | 51992 | 2.85 | |
| Elkhorn Logan Valley Public Health Department | 23461 | 3.41 | 57002 | 3.13 | |
| Three Rivers Public Health Department | 32598 | 4.74 | 77705 | 4.26 | |
| Lincoln/Lancaster County Health Department | 122961 | 17.86 | 285407 | 15.65 | |
| Sarpy/Cass Department of Health and Wellness | 67846 | 9.86 | 184081 | 10.10 | |
| Douglas County Health Department | 189848 | 27.58 | 517110 | 28.36 | |
| Southwest Nebraska Public Health Department | 18061 | 2.62 | 36987 | 2.03 | |
| Two Rivers Public Health Department | 38224 | 5.55 | 94797 | 5.20 | |
| South Heartland District Health Department | 19033 | 2.77 | 46218 | 2.53 | |
| Central District Health Department | 26997 | 3.92 | 75576 | 4.14 | |
| Public Health Solutions District Health Department | 21768 | 3.16 | 55176 | 3.03 | |
| Four Corners Health Department | 17086 | 2.48 | 44216 | 2.42 | |
| Southeast District Health Department | 14844 | 2.16 | 39341 | 2.16 | |
